# High prevalence of hpv multiple genotypes in women with persistent chlamydia trachomatis infection

**DOI:** 10.1186/1750-9378-9-30

**Published:** 2014-09-08

**Authors:** Silva Seraceni, Francesco De Seta, Claudia Colli, Rossella Del Savio, Giuliano Pesel, Valentina Zanin, Pierlanfranco D'Agaro, Carlo Contini, Manola Comar

**Affiliations:** 1 Institute for Maternal and Child Health - IRCCS “Burlo Garofolo” via dell’ Istria 65, 34100 Trieste, Italy; 2 Medical Science Department, University of Trieste, Piazzale Europa 1, 34100 Trieste, Italy; 3 Sexually Transmitted Infection Center, ASS 1, via Gatteri1, 34100 Trieste, Italy; 4 Department of Medical Sciences, University of Ferrara, Via Fossato di Mortara 64/B, 44121 Ferrara, Italy

**Keywords:** Human papillomavirus, *Chlamydia trachomatis*, HPV multiple genotypes, Hsp60 RNA persistent infection

## Abstract

**Background:**

*Chlamydia trachomatis* interaction with HR-HPV types has highlighted a central role in cervical cancer development. The aim of this study was to investigate HPV prevalence and genotypes distribution in women at risk for *C. trachomatis* infection and negative for intraepithelial lesion or malignancy.

**Methods:**

1071 cervical swabs were tested for *C. trachomatis* by Real Time PCR and genotyping by ompA gene sequencing. Additionally, a quantitative Real time-PCR was performed to assess the expression of the *C. trachomatis* Hsp60–encoding gene (Ct604 portion), linked to a persistent status of infection. HPV infection and genotypes was investigated in *C. trachomatis* positive women using Luminex technology.

**Results:**

*C. trachomatis* infection was detected in 53 out of 1071 (4.5%) samples, of which the 53% resulted positive for Hsp60 gene expression. The overall prevalence of HPV infection in *C. trachomatis* positive samples was of 60.4% (32/53): in 37.5% of samples was present a single genotype, while multiple genotypes infections were found in the 62.5% of them. Among women with a *C. trachomatis* chronic infection, 68% were HPV co-infected and the 79% showed multiple genotypes. Should be noted that levels of *C. trachomatis* Hsp60 expression in HPV co-infected women were significantly lower compared to women infected only with *C. trachomatis*. The *C. trachomatis* serotype F was found in the majority of samples, independently of HPV infection.

**Conclusions:**

A high prevalence of HPV multiple infections have been found in young women affected with a *C. trachomatis* chronic infection. These observations suggested that the expression of CHSP60-1, interfering with both apoptotic and cellular senescence pathways, may promote a favourable local microenvironment for HPV infection.

## Background

*Chlamydia trachomatis* (*C. trachomatis*), an intracellular bacteria characterized by a unique biphasic developmental cycle, is the most common sexually transmitted pathogens in women. Although *C. trachomatis* can cause pelvic inflammatory disease (PID), infertility, ectopic pregnancy, the clinical course is usually sub-acute and poorly symptomatic and the microorganism is rarely detected in subjects without clinical signs of infection [1].

The ability of *C. trachomatis* to cause chronic persistent infection, characterized by the permanence of microorganisms in the host cells, can represent a common event [2]. During persistent status, *C. trachomatis* produces a large quantity of Heat shock protein 60 (Hsp60) exhibited on the host cell surface and released into the extracellular space and in the bloodstream. This protein is considered a useful marker during clinical complications since its expression induce host chronic inflammation response [1,3,4]. Thus, this microorganism is considered as a potent immunogen, stimulating a rapid and intense inflammatory response involving previously sensitized lymphocytes [5].

Although epidemiological data have not yet provided consistent evidence about a real implication of *C. trachomatis* in cervical cancer, the co-infection with Human papillomavirus (HPV), sharing the transmission route and the same risk factors, have recently highlighted [6-8]. A role for *C. trachomatis* as cofactor was suggested, since it seems to facilitate the penetration of HPV and the progress of cervical lesions interfering in the immunological response [9]. Moreover, some authors recently detected a high-risk for the development of cervical cancer in patients with HPV infection and history of *C. trachomatis*[10]. Nevertheless, the prevalence and distribution of HPV genotypes associated to *C. trachomatis* infection and its clinical persistence are poorly explored.

HPVs are a family of DNA viruses that infect cutaneous epithelia, oral and genital mucosa. More than 100 different HPV types have been identified and characterized in two risk classes on the basis of their oncogenic potential: Low-risk (LR-HPV) types associated with benign genital warts and High-risk (HR-HPV) type considered the etiological agents of cervical cancer and other genital malignancies [11]. Approximately 15 HR-HPV genotypes are clearly associated with cervical cancer of which HPV16 and HPV18 are the most carcinogenic, since they are responsible for approximately 50% and 20% of all cervical cancers worldwide, respectively. Multiple human papillomavirus genotypes often coexist within cervical epithelia and are frequently detected together in women with precancer cervical lesions [12]. Nevertheless, although HPV is a prerequisite for cervical cancer only a small number of women exposed to this virus developed cancer, implying that other risk factors may be considered as cofactors rather than independent factors. On this basis, the characterization of HPV infection in women suffering from *C. trachomatis* could be important in generating hypotheses regarding the possible synergism of these pathogens in cervical malignancy [13-16].

The aim of this study was to investigate HPV genotypes distribution and the frequency of infection in Italian women considered at risk for *C. trachomatis* infection but negative for cervical lesions or malignancy. Furthermore, the role of *C. trachomatis* chronic infection in promoting HPV susceptibility has been evaluated.

## Methods

### Specimens

In 2013 year, cervical swabs (CS) specimen from 1071 women at risk for *C. trachomatis* infection were collected at the Virology laboratory of the IRCCS-Burlo Garofolo of Trieste, Italy, as part of *C. trachomatis* routine screening practices. Cervical samples were collected using a 200 mm polyethylene Cervex brush device (Rovers Medical Devices B.V., The Netherlands) in 500 μl of TE buffer. The study was approved by the Institutional Scientific Board of the Institute for Maternal and Child Health - IRCCS “Burlo Garofolo”–Trieste, Italy and informed consent was obtained from each participant in accordance with the principles outlined in the Declaration of Helsinki.

### C. trachomatis detection

Genomic DNA was extracted, after samples centrifugation, using the QIAamp DNA Blood miniKit (Qiagen, GmbH, Germany) as indicated by the supplier, and then stored at -80°C until analysis.

The presence of *C. trachomatis* DNA was detected by Real Time PCR (RT-PCR), using a commercial kit (CTDNA, Dia.Pro, Italia), detection limit of the assay was 1 copies/μl, to amplify a conserved region of the cryptic plasmid element of *C. trachomatis*, following recommended protocol. The amplification and PCR product detection were performed with the ABI Prism 7900 Sequence Detection system (Applied Biosystems, Italy).

### HSp60 gene expression

#### *C. trachomatis -RNA extraction and cDNA synthesis*

An aliquot, from each CS fresh specimen (1 ml), was centrifuged at 14.000 rpm for 15 min at 4°C and total RNA was extracted to the pellet obtained by RNeasy Mini kit (Qiagen GmbH, Hilden, Germany) in according to the manufacturer’s instructions. The RNA final concentration (25 μg/ml) eluted in 50 μl of distilled water, was treated during sample processing with Dnase I (RNase-Free DNase Set, Qiagen GmbH, Hilden, Germany), and subsequently stored at -80, in order to avoid DNA genomic contaminations.

cDNA synthesis was performed using kit SuperScript VILO™ cDNA Synthesis Kit (Invitrogen, Carlsbad, CA, USA), according to manufacturer’s instructions. Briefly, 14 μl of RNA were added to a mixture containing: 5X VILO™ Reaction Mix10, 10X Superscript Enzyme Mix and diethyl-pyro carbonate water (DEPC-treated) until a final volume of 20 μl and subsequently incubated at 42°C for 60 minutes and then at 85°C for 5 minutes. cDNA synthesized was employed for quantitative RT-PCR (*q*PCR).

#### *C. trachomatis-Hsp60 qPCR*

To quantify the transcript level for the *C. trachomatis* portion Hsp60 gene (Ct604) in specimen, a dedicated *q*PCR was used as previously described [17,18]. The sensitivity of each assay was determined to be the lowest dilution of DNA (2 10^-4^ ng/ml corresponding to one genome copies/μl) and a standard curve equations were used to calculate the absolute copy number of gene mRNA.

In brief, the test included a serial standard curve, negative control with PCR-grade water and a positive control (*C. trachomatis* strain-TW-3). PCR reaction, in a final volume of 20 μl, contained: 5 μl of cDNA, 2 μl LC FastStart DNA Master SYBR Green I (Roche Molecular Biochemicals, Germany), 5 mM MgCl_2_ and 0.8 μM each primer. The thermocycling condition was: 95°C at 10 min followed by 40 cycles of 15 s at 95°C and 1 min at 63°C. The amplification was carried out in an ABI 7900HT Fast Real Time PCR System (Applied Biosystems, Italy). The concentration of unknown samples based on their *C*t values was determined with analytical software (Software SDS 2.4; Applied Biosystems) [19]. The specificity of *q*PCR was further confirmed by agarose gel electrophoresis analysis, which showed the expected amplification product of 161 bp in length.

#### *C. trachomatis genotyping*

*C. trachomatis* genotyping were performed by ompA gene primers; positive PCR amplification products were purified, sequenced and analysed with BLAST program (http://www.ncbi.nlm.nih.gov/BLAST) as previously described [20].

#### *HPV detection and characterization*

HPV was detected in CS specimens using molecular assay supported by the Luminex technology (Luminex Corporation, Austin, TX). HPV genotyping was performed using the type specific E7 polymerase chain reaction bead-based multiplex assay (TS-E7-MPG, IARC, Lyon, France) as recently described [21]. The detection limits of the assay ranged from 10 to 1,000 copies of the viral genomes included in the analysis. In addition, the β-globin gene was included, as internal positive control [22]. To analyse a greater number of LR-HPV types, (HPV-6,-11,-40,-42,-43,-44,-54,-61,-70), the Anyplex™ II HPV Detection assay (Seegene Inc., Arrow diagnostics, Italy) was additionally used in according to manufacturer’s instructions. A human housekeeping gene was used as an endogenous internal control, which can ensure DNA purification, PCR reaction and specimen quality (Anyplex™ user manual, Seegene 2012).

### Statistical analysis

Chi Square Test was used to compare frequencies of discrete variables: Fisher Exact Test was applied when necessary. P value ≤ 0.05 was considered as the threshold of statistical significance for all tests.

## Results

During 2013 year, 1071 cervical swabs from women at risk for *C. trachomatis* infection (mean age 35 ± 10 years; range: from 15 to 72 years) were analysed at the Virology laboratory of IRCCS-Burlo Garofolo, Trieste, Italy. Of these, 829 included Outpatients (mean age 35 ± 10 years), 161 patients from Sexually Transmitted Infection centre (STI) (mean age 30 ± 10 years) and 81 women from the Assisted Reproductive Technology (ART) (mean age 37 ± 10 years) clinic. All women were asymptomatic for *C. trachomatis* and other genital infections at the time of sampling, with the exception of STI women showing inflammatory symptoms. Moreover, all women were negative for cytological alterations, in accordance with Bethesda System 2001 diagnostic criteria [23].

In this study, as showed in Table 1, the overall prevalence of *C. trachomatis* infection was 4.5% (53/1071) (mean age 35 years). As expected, *C. trachomatis* prevalence, stratified by the different clinical departments and by age, was found statistically significant higher (12.4%) in symptomatic women attending the STI center, than in asymptomatic women from the other groups (p < 0.001).

**Table 1:** Prevalence of CT distribution in women at risk of infection by clinical department and mean age

**Clinical Department**	**n° women**	**Mean Age ± ơ**	**CT n° + (%)**
**Outpatients**	829	35 ± 10	29 (3.5%)
**STI**	161	30 ± 10	20 (12.4%)
**ART**	81	37 ± 10	4 (4.9%)
**Total**	1071	35 ± 10	53 (4.5%)

**STI:** Sexually Transmitted Infection; **ART:** Assisted Reproductive Technology; **CT:***C. trachomatis.*

**Figure 1:**
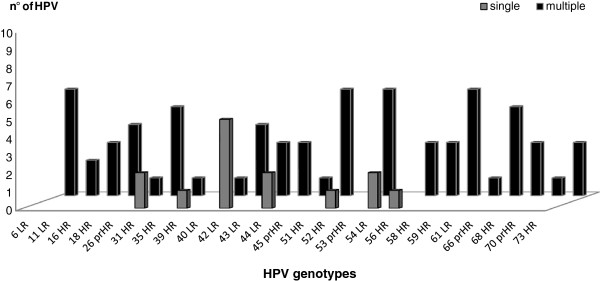
**HPV genotypes distribution detected in *C. trachomatis *positive women, according to single versus multiple concurrent infection status.** HR: High risk; prHR: Presumable High risk; LR: Low risk.

**Figure 2:**
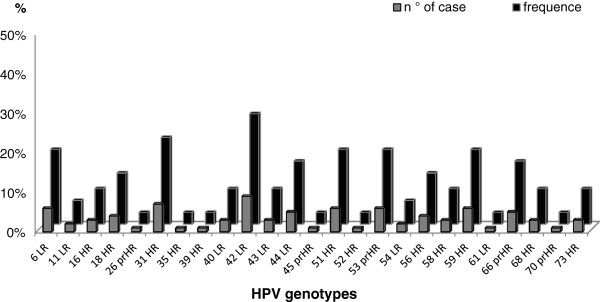
**Prevalence of HPV genotypes detected in *C. trachomatis *co-infected women (n° and%).** HR: High risk; prHR: Presumable High risk; LR: Low risk.

In women with a *C. trachomatis* infection the overall prevalence of HPV was high, tested to 60.4% (32/53), as shown in Table 2. Regarding the distribution of HPV, the 37.5% (12/32) of the infections were constituted by a single genotype while the 62.5% (20/32) by multiple genotypes (from 2 to 8 types), recovered more frequently in younger women (mean age 24 years) (Figure 1).The analysis of HPV genotypes, reassumed in the Figure 2, showed that HPV-42 and HPV-31 represented the most frequently detected genotypes, standing at 28% and 22%, respectively. Moreover, in these women, the genotypes HPV-39,-53,-56,-58 were present only as single infection while the genotypes HPV-6-51-59-66 were detected together in 31% (10/32) of the recovered infections.

**Table 2:** HPV co-infection distribution in women with CT infection

**CT POS**	**HPV**	**HPV single infection**	HPV multiple infections*
**N°**	**N°POS/TOT**	**N°POS/TOT**	**N°POS/TOT**
	**(%)**	**(%)**	**(%)**
53	32/53	12/53	20/53
	(60.4)	(22.6)	(37.7)

*(from 2- to 8 genotypes); **CT:***C. trachomatis;***HPV:** Human papillomavirus.

In order to characterize the chlamydial phase of infection, the mRNA expression of the Hsp60 gene showed that the 53% (28/53) of the women with *C. trachomatis* were suffering from a chronic infection (Table 3). Of these women (mean age 26 years), the 68% (19/28) resulted co-infected with HPV. In particular, the 79% (15/19) presented multiple infections and the 21% (4/19) single infections. Moreover, the level of Ct-Hsp60 expression was found significantly lower (±396 copy/μl) in women co-infected with HPV compared to women infected only with *C. trachomatis* (±862 copies/μl). The evidence for viable organisms and not just residual DNA from a previous infection was supported to high correlation between DNA and RNA results (data not shown), considering that, the expressed gene should be linked to the *C. trachomatis* persistence.

**Table 3:** **RNA expression of the CT *- *** **Hsp60 gene in relation to the presence or absence of HPV infection**

	**CT-Hsp60 RNA**	
	**(+)**	**(-)**
**N°/TOT (%)**	28/53 (53)	25/53 (47)
**HPV + n° (%)**	19/28 (68)*	13/25 (52)
**HPV- n° (%)**	9/28 (32)	12/25 (48)

*4/19 (21%) HPV single infection and 15/19 (79%) HPV multiple infections.

**CT:***C. trachomatis*; Hsp60: Heat shock protein 60; HPV: Human papillomavirus.

The classification of *C. trachomatis* serotypes through the ompA gene amplification and subsequent sequencing, performed in available samples, had revealed a high frequency of serotype F, independently of chlamydial status or HPV infection.

## Discussion

Several epidemiological studies have stated a positive association between *C. trachomatis* and HPV-related cervical diseases. The co-presence of *C. trachomatis* and HPV was reported in cervical precancerous lesions, and high levels of specific IgG antibodies or DNA of *C. trachomatis* were recovered in HPV positive patients [6,14,15]. However, the exact relationship between *C. trachomatis* and HPV infection is still not completely understood.

In the present analysis, we evaluated the distributions of HPV DNA-positive infections analysing a large number of specimens collected as part of routine screening practices for *C. trachomatis* prevention. For the first time, estimates are provided on the prevalence of HPV infections and about genotype distribution in women with a chronic *C. trachomatis* infection; this work has never been assessed before. Data from our study showed that the 60.4% of women with a diagnosis of *C. trachomatis* infection and in absence of cervical lesions, resulted co-infected with one or more HPV genotypes. To note, the 53% of them showed a chronic infection and, HPV was found more frequently associated (68%) to this specific chlamydial status. Moreover, a consistent portion of these women (79%) resulted to be infected with multiple HPV genotypes. To note, HPV multiple infections including specific genotypes such as HPV-6-51-59-66, was reported in the 31% of these women supporting evidence that the presence of one HPV type does not increase the likelihood of acquiring further infections, but that, HPV multiple infections might be the result of the local immune system impairment [8,24-31]. In our screening population, the median age of women with 2 to 8 HPV genotypes was 24 years, according to recent data [32,33].

In our series, HPV-31 and HPV-42 represented the genotypes more frequently detected, testing at 28% and 22%, respectively. The overall prevalence of these genotypes, with a smaller estimated oncogenic potentials than HPV-16, were usually lower in HPV screening Italian women (13.8% and 0.3%,) [34,35]. While, conversely, HPV-16 (30.7%) [34] was found only in the 9%.

In the natural history of *C. trachomatis*, a chronic infection is referred as a stop in development of chlamydial cycle, with aberrant bodies formation, and this state is characterized by high transcriptional activity [36].

In this study, the expression of Hsp60 gene, a marker of chronic infection, has been found significantly lower in HPV co-infected women compared to women infected only with *C. trachomatis.* Discussion on this finding can be merely speculative, suggesting that the maintenance of a steady-state level of transcription of Hsp60 gene could favour a balance between the Hsp60 induced pro-inflammatory microenvironment and HPV coexistence [14].

The chronic inflammation caused by *C. trachomatis* increases oxidative stress proteins that seem to trigger HPV cell entrance, and replication or enhance DNA breaks that may promote viral integration [9,14,16]. Although it has been suggested that the concomitant presence of HPV viral oncoproteins during Hsp60 expression may lead to the ability to survive apoptotic stimuli, uncontrolled proliferation and, finally neoplastic transformation, in this study specific HPV multiple genotypes were found associated to *C. trachomatis* chronic status, independently of the genotypes risk [37,38].

## Conclusions

In conclusion, the results of the present investigation provide evidence for the notion that a high prevalence of multiple HPV infections has been associated with *C. trachomatis* chronic infection in young women without cervical lesions. In addition, specific HPV genotypes seem to be more frequently associated to *C. trachomatis.* This data may deserve further consideration, owing to accumulate evidence that the chlamydial chronic status could contribute to favour specific HPV genotypes representing possible implications for the prevention of cervical cancer.

## Abbreviations

HPV: Human papilloma virus; LR-HPV: Low risk-HPV; HR-HPV: High risk-HPV; C. trachomatis: Chlamydia trachomatis; CIN: Cervical intraepithelial neoplasia; ICC: Invasive cervical carcinoma; PID: Pelvic inflammatory disease; Hsp60: Heat shock protein 60; CS: Cervical swabs.

## Competing interest

The authors declare that they have no competing interests.

## Authors’ contributions

SS contributed to: study concept and design, acquisition of data (Chlamydia molecular and CT-Hsp60), drafting of the manuscript. FDS and CC contributed to: collection of clinical specimens and demographic data. RDS and VZ contributed to: technical assistance in molecular HPV analysis. GP contributed to: data analysis. PDA contributed to: data analysis. CC contributed to: drafting of the manuscript. MC contributed to: study concept and design, interpretation of data, and revising the manuscript. All authors read and approved the final manuscript.
